# Inhibition, shifting and updating in relation to psychometric intelligence across ability groups in the psychiatric population

**DOI:** 10.1111/jir.12559

**Published:** 2018-11-07

**Authors:** K. E. Biesmans, L. van Aken, E. M. J. Frunt, P. A. M. Wingbermühle, J. I. M. Egger

**Affiliations:** ^1^ Specialist and Forensic Care STEVIG Oostrum The Netherlands; ^2^ Centres of Excellence for Neuropsychiatry and Korsakoff Vincent van Gogh Institute for Psychiatry Venray The Netherlands; ^3^ Donders Institute for Brain, Cognition and Behaviour Radboud University Nijmegen The Netherlands

**Keywords:** contextual neuropsychology, executive function, intellectual disability, IQ, neuropsychiatry

## Abstract

**Background:**

Assessment of intelligence and executive function (EF) is common in complex neuropsychiatric practice. Although previous studies have shown that EF and intelligence are related, it is unknown whether these constructs relate to one another in a similar manner across different ability groups (mild intellectual disability, borderline intellectual disability and normal/high intelligence). This study therefore examines the relation between three EFs (inhibition, shifting and updating) and intelligence in a heterogeneous psychiatric sample. It is hypothesised that the strength of the relation between intelligence and the three EFs decreases when the level of intelligence increases, in accordance with Spearman's Law of Diminishing Returns.

**Methods:**

In a cross‐sectional, between and within subject design, one of the three intelligence tests (Kaufman Adolescent and Adult Intelligence Test and Wechsler Adult Intelligence Scale – third and fourth editions) and several EF tests (Stroop Colour–Word Test, Trail Making Test and Spatial Working Memory task) were administered to 250 neuropsychiatric inpatients and outpatients (M_age_ = 39.8, standard deviation = 14.3, 52.8% male). Based upon their full‐scale IQ score, patients were divided into three ability groups (mild intellectual disability, borderline intellectual disability or normal/high intelligence). The relation between EF and intelligence was assessed through analyses of the correlation pattern; groups were compared using analysis of covariance.

**Results:**

Analyses showed significant correlations between the constructs of EF and intelligence. A significant interaction effect was found for shifting, with highest correlations in the normal to high intelligence group, but not for inhibition and updating.

**Conclusions:**

Results support a specific role for shifting in this EF–intelligence relation. The correlational pattern of updating and intelligence, as well as the differential relation of shifting and intelligence across ability groups, suggests that EF tasks may not measure distinct EFs in lower intellectual ability but rely on cognitive primitives such as processing speed. EF tasks can be considered less valid indicators of EF ability. Implications in terms of the need for development of specific tasks to measure cognition in low intellectual ability are discussed.

## Introduction

Executive function (EF) refers to the complex control mechanisms that enable a person to optimally use one's cognitive abilities in order to perform effective, goal‐directed and self‐regulating behaviour (Lezak *et al*. 2004; Salthouse 2005; Barkley 2012; Roelofs *et al*. 2015). While there is great variety in defining EF, the following global areas can be identified: attention, emotion regulation, flexibility, inhibitory control, initiation, organisation, planning, self‐monitoring and working memory (WM) (Goldstein *et al*. 2014; Goldstein & Naglieri 2013).

The concept of EF originates from early reports of patients with prefrontal damage (Stuss & Benson 1986) and is embedded as a key element in both neuropsychological information processing models as well as in patient care (van Aken 2017). Theoretical models of EF have evolved around the fundamental question whether executive processes can be explained by one single underlying ability or whether these components are distinct, but related processes (Jurado & Rosselli 2007), challenging the conceptualisation and operationalisation of the construct of EF. Using factor analysis, Miyake *et al*. (2000) presented a model that identifies three separable but partially correlated constructs: inhibiting prepotent responses (inhibition), shifting between tasks or mental sets (shifting) and updating of WM representations (updating). In contrast, Packwood *et al*. (2011) reviewed the literature and identified over 30 executive subcomponents, emphasising the great conceptual diversity and operational difficulties of EF.

Intelligence can be defined as a general capacity that, among other things, involves the ability to reason, plan, solve problems, think abstractly, comprehend complex ideas, learn quickly and learn from experience (Gottfredson 1997a). It is not merely book learning, a narrow academic skill or test‐taking smarts. Rather, it reflects broader and deeper capability for comprehending our surroundings (Gottfredson 1997b). More than a century ago, Spearman proposed a general factor (*g*) reflecting a cognitive ability that is applicable to any kind of cognitive problem. This *g* factor is assumed to contribute to success in diverse cognitive ability tasks, showing a pattern of positive correlations known as *the positive manifold* (Spearman 1904). Horn and Cattell (1966) divided general intelligence (*g*) into crystallised intelligence (Gc) and fluid intelligence (Gf), which also are the fundamental abilities in the updated and more expanded Cattell–Horn–Carroll theory of cognitive abilities (Schneider & Mcgrew 2012). The relation between Gf and Gc is described in Cattell's investment theory, stating that crystallised knowledge expands through the investment of fluid abilities, describing the process of learning (Cattell 1957; Cattell 1963; Schweizer & Koch 2002).

Looking at definitions of EF and intelligence, it follows that they strongly relate on a conceptual level. Both include the ability to successfully adjust to new situations (Lezak *et al*. 2004; Miyake *et al*. 2000; Diamond 2013) and are strongly predictive for the success in many real‐world activities like educational attainments, social mobility and job performance (Gottfredson 1997b). Various studies examined how different EFs are interrelated and how they are related to both *g* and Gf. In particular, the construct of EF was found to be closely related to established cognitive abilities of Gf and processing speed (Arffa 2007; Floyd *et al*. 2010; Godoy *et al*. 2014; Salthouse 2005; Salthouse *et al*. 1998; Salthouse & Davis 2006; Salthouse & Pink 2008), and especially WM seems to be closely related to Gf (Arffa 2007; Carpenter *et al*. 1990; Chuderski 2013; Diamond 2013; Duggan & Garcia‐Barrera 2015; Duncan *et al*. 2012; Floyd *et al*. 2010; Godoy *et al*. 2014; Friedman *et al*. 2006; Miyake *et al*. 2001; Redick *et al*. 2012; Salthouse 2005; Salthouse *et al*. 2003; Salthouse *et al*. 1998; Salthouse & Pink 2008; Schneider & Mcgrew 2012; van Aken *et al*. 2015).

An attempt to integrate information processing theories and the psychometric concept of intelligence has been made by Duncan (2013). Based on human functional brain imaging, he proposed a multiple‐demand (MD) system, a system that is involved in novel and complex cognitive challenges in many different domains, for example, perception, response selection, language, memory or problem solving. According to Duncan (2013), the MD system can be seen as the neural basis of Gf, describing intelligence as the efficiency with which novel or complex tasks are solved. Gf operates as a general, domain‐independent process involved in various cognitive tasks. Duncan suggests a strong relation between Gf and EF, implying that cognitive tasks measuring specific domains (e.g. EF tasks) always contain a Gf component.

Spearman's Law of Diminishing Returns (SLODR; Spearman 1904; Spearman 1927) may further contribute to understanding the relation between intelligence and EF. It states that individuals with lower mental abilities show more *g*‐loadings and less differentiated cognitive profiles than individuals with higher mental abilities. Furthermore, *g‐*loadings of cognitive ability tests are expected to decrease as a function of ability or age, indicating higher *g‐*loadings for low ability or young groups (Saklofske *et al*. 2008). This suggests that – in individuals with lower mental abilities – performances on cognitive tasks can be fully explained by *g* instead of by specific, domain‐dependent cognitive processes. Nonetheless, numerous studies replicated findings indicating separate and distinguishable EFs in independent samples (Friedman & Miyake 2017), including a sample of individuals with mild to borderline intellectual disability (ID) (Roelofs *et al*. 2015). This may suggest that both domain‐independent and domain‐dependent cognitive processes exist throughout the intellectual ability range, although these processes may relate differently to one another.

In the (neuro)psychiatric population, a high prevalence of premorbid intelligence deficits or ID is found (Stratta *et al*. 2008; Verhoeven & Egger 2014), and research on gene–environment synergism already offers valuable contributions to psychiatric epidemiology. Several key risk factors of neuropsychiatric disorders (e.g. schizophrenia, bipolar disorder, affective disorder, autism or attention deficit hyperactivity disorder) are being documented, such as paternal age, psychiatric family history, neonatal vitamin D deficiency or socio‐economic adversity (Pedersen *et al*. 2018). Level of intelligence also seems to be a causal factor in psychiatric illness, showing people with ID of all ages to show substantially more health problems (e.g. physical disability, learning disability or developmental disorders), poorer general health (Hughes‐Mccormack *et al*. 2017) and high risk of polypharmacy (Bratek *et al*. 2017).

Regarding cognition, there are indications that people with ID often show EF deficiencies (Danielsson *et al*. 2010; Willner *et al*. 2010). Danielsson *et al*. (2010) showed that people with ID perform below chronologically aged‐matched peers on a range of EF tests. When compared with mentally aged‐matched peers, however, people with ID performed equivalent. Also, over a 5‐year period, no significant changes in EF performances were found between adults with ID and controls. They did find that, compared with normal controls, individuals with ID had no difficulties in the area of non‐verbal planning or inhibition but show a significant impairment in the area of shifting. Other studies show similar capacity but problems in actively manipulating stored information (compared with mentally aged‐matched controls) (Carretti *et al*. 2010; Numminen *et al*. 2002). A syndrome study including participants with Prader‐Willi and Fragile X syndromes, examining the association between repetitive behaviours and deficitis in EF, found similar results. Specifically, deficits in EF task switching were found in the Prader‐Willi group, when compared with normal controls (Woodcock *et al*. 2009).

Examining the relation between EF and *g* across ability groups is important, because EF performance might depend on the level of intellectual ability itself. Unravelling this interplay of EF and *g* (and its relevance for our understanding of domain‐independent and domain‐dependent cognitive processes) can lead to more differentiated and focalised neuropsychological assessment and intelligence testing.

Hence, the purpose of the present study is to determine the relation among three EFs (inhibition, shifting and updating) and *g* across ability groups in the psychiatric population. It is hypothesised that the EF measures for inhibition, shifting and updating relate differentially to intelligence across different ability groups. According to Spearman's law (SLODR; Spearman 1904; Spearman 1927), the EF and intelligence correlations are expected to be strong in the low ability group and to decrease when the level of intelligence increases. More differentiated (and less *g*‐loaded) EF profiles are to be expected in the normal to high intelligence group.

## Method

### Participants

Included were 250 neuropsychiatric inpatients and outpatients in the age of 16 to 80 years [M_age_ = 39.8, standard deviation (SD) = 14.3, 52.8% male] of the Vincent van Gogh Institute for Psychiatry in Venray, the Netherlands. Participants were divided into three ability groups. The mild ID group (*n* = 32; M_age_ = 39.4, SD = 11.8, 46.9% male) contained patients with a full‐scale IQ (FSIQ) score in the range of 50 to 70, the borderline ID group (*n* = 78; M_age_ = 40.1, SD = 13.7, 48.7% male) contained patients with an FSIQ score in the range of 71 to 84 and the normal/high intelligence group (*n* = 140; M_age_ = 39.7, SD = 15.2, 56.4% male) contained patients with an FSIQ score in the range of 85 to 130. No exclusion criteria – other than age under 16 years – were used. Data were collected between March 2006 and June 2016, as part of standard neuropsychological assessment. Included were patients with complex, multiple diagnoses ‐ according to the *Diagnostic and Statistical Manual of Mental Disorders, Fourth Edition Text Revision* ‐ who were referred in the context of psychopharmacological and/or psychological treatment. Test results were drawn from an electronic database; patient identities were concealed. Because this study is part of an ongoing research project on the assessment of neuropsychiatric patients, some of the included patients were also enrolled in studies of van Aken (2017).

### Materials

The tests that measure EF were selected following the model of Miyake *et al*. (2000).

#### Inhibition

The Stroop Colour–Word Test (Stroop 1935; Van der Elst *et al*. 2006) was administered as a measure of response inhibition (through selective attention and cognitive flexibility). In the first condition, participants need to read words (names of colours) out loud. In the second condition, participants need to name displayed colours. In the third and key condition, participants need to suppress the dominant tendency to read the written words (names of colours) and instead name the incongruent font colour. The response time on this incongruent condition (card III) was used for analyses.

#### Shifting

The Dutch language version of the Trail Making Test (D‐KEFS TMT; Delis *et al*. 2001) requires participants to switch back and forth between sequencing numbers and letters. It measures the ability of set shifting and calls on executive attention. The first, second, third and fifth conditions of the TMT measure basic skills that are required for set shifting (respectively visual scanning, number sequencing, letter sequencing and motor speed). The response time on the shifting condition (fourth condition) was used for analyses.

#### Updating

All participants completed the Spatial Working Memory task from the Cambridge Neuropsychological Test Automated Battery (Robbins *et al*. 1998; Lowe & Rabbitt 1998; Potvin *et al*. 2005). In this task, participants touch boxes in order to find a blue token (using a process of elimination). The number of boxes gradually increases, up to a total of eight boxes. Spatial Working Memory task capacity is reflected by the number of between errors (searching tokens in boxes that have been opened before).

#### Intelligence

The FSIQ scores were derived from three intelligence test batteries: the Kaufman Adolescent and Adult Intelligence Test (KAIT, *n* = 106; Mulder *et al*. 2005) and the Dutch language versions of the Wechsler Adult Intelligence Scale – third and fourth editions (WAIS‐III, *n* = 28; and WAIS‐IV, *n* = 118) (Wechsler 2012a; Wechsler 2012b). The FSIQ scores were included for analyses.

The KAIT contains six subtests, which make up a crystallised IQ scale, a fluid IQ scale and the FSIQ. The subtest reliabilities (Cronbach's α) vary between 0.78 and 0.91 (Mulder *et al*. 2005). The Dutch versions of the WAIS‐III and WAIS‐IV both contain 10 core subtests and, respectively, two and five optional subtests. Secondary to the FSIQ, scores on four indices (verbal comprehension index, perceptual organisation index, WM index and processing speed index) are provided in the WAIS‐III. These indices were theoretically and psychometrically enhanced in the revision of the WAIS‐III to WAIS‐IV, along with the elimination of the verbal and performal IQ scales. The indices remained similar in terms of abilities that are called on, except for the perceptual organisation index. This index was transformed into the perceptual reasoning index, incorporating more aspects of fluid intelligence. Reliability statistics of the WAIS‐III range from 0.72 for the subtest picture arrangement to 0.93 for the subtest vocabulary (Wechsler 2005). The split‐half reliability of the subtests and indexes of the WAIS‐IV varies from 0.75 to 0.97 (Wechsler 2012a; Wechsler 2012b).

### Procedures and analyses

Each participant completed one intelligence test and all executive tests, which were administered and scored by qualified psychologists according to the test manuals. Test sessions generally took place in two sessions of approximately 3 h of testing time each. The data were analysed using ibm spss statistics 22 (IBM Corp 2013). Extreme values (>5 SD) were identified in 11 cases and were removed.

The FSIQ scores of the participants were merged into one variable. The three variables regarding EF (inhibition, shifting and updating) were reversed in order to obtain positive relations and facilitate interpretation. Moreover, a composite EF score was computed by deriving the mean of the standardised scores on inhibition, shifting and updating for each case, as a unitary representation of the construct of EF.

Levene's test on homogeneity of variance was performed for FSIQ and the EF variables to assess the equality of variance in the ability groups. Fisher r‐to‐z transformation was conducted in order to test whether the correlations between the three EF measures and psychometric intelligence differ across the ability groups. Subsequently, a one‐way between‐group analysis of covariance (ANCOVA) was conducted in order to examine the possible moderating effect of group in the relation between the composite EF score and FSIQ (Field 2013). The independent variable was group, the dependent variable was FSIQ and the standardised composite EF score was added as a covariate. The interaction effect of group*composite EF score was added to the model. Subsequently, separate ANCOVAs were conducted for the three separate EFs. The independent variable remained group, and the dependent variable remained FSIQ; the EF measures (respectively inhibition, shifting and updating) were added as covariates, and interaction terms were added to the models. A one‐way between‐group analysis of variance was conducted in order to assess not just if but also the way in which group moderates the relationship between EF and intelligence.

## Results

Table 1 shows the descriptive statistics of the performances on the intelligence test and the EF tasks per group and for the entire sample.

**Table 1:** Mean performance scores, SDs and range of scores per group and for the sample

Measure	MID group			BID group			Normal/high intelligence group			Sample		
	M	SD	Range	M	SD	Range	M	SD	Range	M	SD	Range
Inhibition	132.5	29.4	85–197	114.9	36.7	64–273	100.1	32.9	54–244	108.8	35.4	54–273
Shifting	102.2	42.5	38–192	71.2	29.5	25–198	57.8	28.1	17–185	67.6	33.9	17–198
Updating	42.1	18.1	14–71	33.1	18.7	0–78	25.3	19.5	0–92	29.9	19.9	0–92
FSIQ	63.2	4.1	54–70	78.1	3.7	71–84	99.3	10.8	85–127	88.1	15.9	54–127

FSIQ, full‐scale IQ; MID, mild intellectual disability; BID, borderline intellectual disability; SD, standard deviation.

The values of inhibition and shifting are completion time of the task; the values of updating are number of errors.

Results of Levene's test on homogeneity of variance showed that equality of variance can be assumed for the composite EF score, inhibition and updating but not for shifting and FSIQ.

Table 2 shows the Pearson product–moment correlation coefficients of the relationships between EF performances and FSIQ in the entire sample. All of these correlations are statistically significant and vary in strength from medium to large.

**Table 2:** Pearson product–moment correlations between executive functions and FSIQ (*N* = 250)

	Composite EF	Inhibition	Shifting	Updating	FSIQ
Composite EF	—				
Inhibition	0.770** [0.707, 0.823]	—			
Shifting	0.843** [0.795, 0.882]	0.521** [0.413, 0.616]	—		
Updating	0.748** [0.687, 0.808]	0.296** [0.154, 0.407]	0.469** [0.366, 0.559]	—	
FSIQ	0.502** [0.396, 0.593]	0.370** [0.235, 0.484]	0.478** [0.368, 0.567]	0.336** [0.218, 0.444]	—

EF, executive function; FSIQ, full‐scale IQ.

BCa bootstrap 95% confidence intervals reported in brackets.

** *P* < 0.001 (two tailed).

The composite EF score and FSIQ share 25.2% of their variance (*R* ^2^ = 0.252). The shared variance of the separate EFs (inhibition, shifting and updating) and FSIQ is, respectively, 13.7%, 22.8% and 11.3% (*R* ^2^ = 0.137, *R* ^2^ = 0.228 and *R* ^2^ = 0.113).

Table 3 shows the Pearson product–moment correlation coefficients of the relationship between FSIQ and EF performance within the groups. No group differences were found in correlations between respectively inhibition, shifting updating and psychometric intelligence (*z*‐scores all < 1.88, *P*‐values all > 0.05).

**Table 3:** Pearson product–moment correlations between executive functions and FSIQ within the groups

Group		Composite EF	Inhibition	Shifting	Updating
MID group (*n* = 32)	FSIQ	0.159 [−0.252, 0.566]	−0.104 [−0.430, 0.240]	0.190 [−0.167, 0.504]	0.194 [−0.228, 0.575]
BID group (*n* = 78)	FSIQ	0.278* [0.069, 0.472]	0.172 [−0.037, 0.433]	0.278* [−0.048, 0.468]	0.171 [−0.045, 0.365]
Normal/high intelligence group (*n* = 140)	FSIQ	0.339** [0.184, 0.487]	0.270** [0.094, 0.441]	0.356** [0.200, 0.494]	0.190* [0.035, 0.337]

EF, executive function; FSIQ, full‐scale IQ; MID, mild intellectual disability; BID, borderline intellectual disability.

BCa bootstrap 95% confidence intervals reported in brackets.

** *P* < 0.001 (two tailed).

* *P* < 0.05 (two tailed).

**Figure 1:**
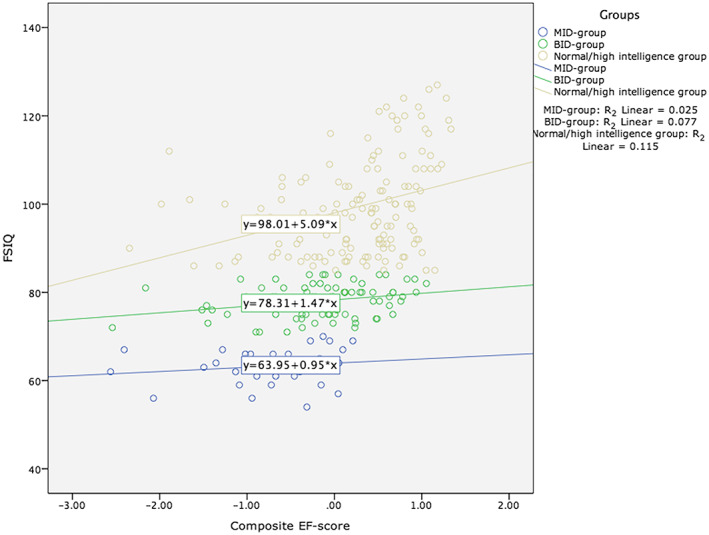
Scatterplot of the composite executive function (EF) score and full‐scale IQ (FSIQ) for the following groups. Mild intellectual disability (MID) group: R_2_ linear = 0.025; borderline intellectual disability (BID) group: R_2_ linear = 0.077; normal/high intelligence group:. [Colour figure can be viewed at wileyonlinelibrary.com]

A significant interaction effect was found for the composite EF score, *F*_2,244_ = 3.31, *P* = 0.038, *η* ^2^_p_ *=* 0.026. Figure 1 shows the scatterplot of the composite EF score and FSIQ for the three groups. Subsequently, the three separate ANCOVAs, which were focused on the three separate EF domains, demonstrated a significant interaction effect for group*shifting, *F*_2,242_ = 4.71, *P* = 0.010, *η* ^2^_p_ *=* 0.038, but not for inhibition and updating.

**Figure 2:**
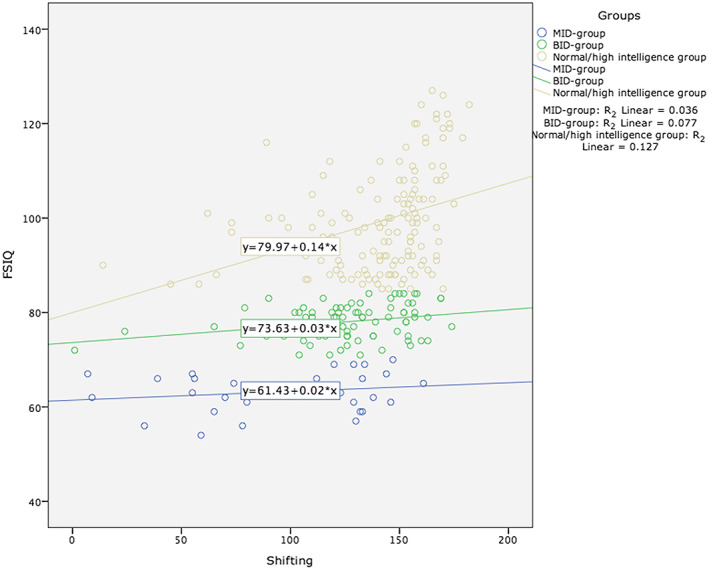
Scatterplot of shifting and full‐scale IQ (FSIQ) for the following groups. Mild intellectual disability (MID) group: R_2_ linear = 0.036; borderline intellectual disability (BID) group: R_2_ linear = 0.077; normal/high intelligence group: R_2_ linear = 0.127. [Colour figure can be viewed at wileyonlinelibrary.com]

Figure 2 shows the scatterplot of shifting and FSIQ for the three groups.

A one‐way analysis of variance only showed a statistically significant relationship between shifting and FSIQ in the normal/high intelligence group (*P* = 0.004). Because the three EF measures were used in the analysis, a Bonferroni correction was applied.

## Discussion

The present study examined whether the relation between EF and psychometric intelligence varies over three groups of psychiatric patients with a different level of intellectual ability, ranging from disabled to high. As hypothesised, the composite EF score relates differently to intelligence across ability groups, with higher correlations in the normal to high ability group. Because the correlational pattern of inhibition and updating was equal across ability groups, shifting explains most of the correlational pattern of the composite EF score.

Correlational analysis indicates EF and intelligence to be related but separable constructs. This is in line with previous research indicating the existence of separable and distinguishable EFs in individuals with mild to borderline ID (Roelofs *et al*. 2015). Current results also support Miyake's view of EF being both unitary and diverse (Friedman & Miyake 2017), because the differential relation between EF and intelligence across ability groups is mostly accounted for by shifting, independent of looking at EF as unitary or diverse.

The current finding of increasing correlations in higher ability groups for shifting (and inhibition showing such an upward trend) is, however, not in line with our hypothesis based on SLODR (Spearman 1904; Spearman 1927; Saklofske *et al*. 2008). Nonetheless, results are similar to the findings of Fogarty and Stankov (1995), Hartmann and Teasdale (2004) and Hartmann and Teasdale (2005), also showing an upward trend between EF and intelligence. Other previous findings suggest no differences in *g‐*loadings on cognitive tasks for different ability groups (Facon 2004; Fogarty & Stankov 1995; Hartmann & Reuter 2006).

According to both SLODR and Duncan's MD system (Duncan *et al*. 2012), less *g‐*loadings and more cognitive differentiation would be expected as levels of intelligence increase across ability groups, suggesting a differentiation of domain‐independent versus domain‐dependent cognitive functions in the normal/high ability group.

Complex cognitive tasks measuring executive attention (divided and sustained attention, goal maintenance/set shifting), response inhibition or cognitive flexibility rely on underlying *cognitive primitives* such as basic (visual) processing speed, visuospatial perception, visual search strategy and visual scanning. Particularly in a sample of people with ID, as used in the current study, results on EF tasks can be considered less valid indicators of EF ability. For instance, a performance on a shifting task measuring divided attention and cognitive flexibility may not only represent the cognitive demand of switching (requiring mental tracking, extra WM capacity, focused attention and set shifting). Several underlying cognitive processes can be accountable for slower reaction times and a higher number of mistakes on this task (e.g. problematic processing of semantic information, problematic sustained attention and lowered processing speed). This also applies to our results from the inhibition condition, which show heightened interference scores that may be caused by a lowered processing speed score instead of a specific deficit in response inhibition (Bouma *et al*. 2012; Stapert *et al*. 2012; Bouma *et al*. 2012). Performances on the Stroop task may also be confounded by the effects of intelligence and educational level (Homack & Riccio 2004), and insufficient reading dominance. A study of Golden and Golden (2002) compared the Stroop performances of children with learning, psychiatric and attentional disabilities with those of healthy controls. Children with learning disorders appeared to be slower on all three basic measures and showed less interference (as would be expected based on the reading dominance theory). Children with attention deficit hyperactivity disorder displayed normal reading capabilities but impaired interference capabilities because of problems in attentional or EFs. Besides the underlying cognitive primitives, task impurity is also a problem concerning EF assessment in all intellectual ranges (Miyake *et al*. 2000; van Aken 2017). As discussed before, no part of the brain works by itself, so current neuropsychological tasks do not purely call on one specific executive process (Luria 1973). In case of deficits in one or more of these non‐specific cognitive abilities, the performance on the executive tasks does not purely represent the supposedly underlying (single) cognitive ability. Therefore, performances on EF tasks may be influenced predominantly by processing speed, especially in a population of people with ID.

Previous studies focusing specifically on low intellectual ability and co‐morbid developmental disorders or psychiatric conditions found similar results on cognitive primitives, indicating difficulties in validity in this group and therefore interpretation of EF measures in a population of people with ID. For example, Danielsson *et al*. (2010) found shifting deficits in low intellectual ability as a result of problematic executive control. Carretti *et al*. (2010) stressed the importance of active attentional control in explaining the role of WM in fluid intelligence performance. Anderson (1992) proposed that, at lower ability levels, performance is essentially determined by basic processing speed, whereas at higher ability levels/processing speed, differences in specific processors related to particular tasks should become more apparent. This relates to current results. The updating task used in our study was the only task that did not entail the element of *processing speed*, resulting in equal correlations with intelligence in the all ability groups.

Some limitations of the current study need to be discussed. First, a heterogeneous sample was used, in which a broad spectrum of psychiatric conditions, intellectual functioning and neuropsychological deficits is represented. The performance of patients is therefore influenced by multiple interfering factors that differ across individuals (e.g. the nature and the severity of the psychiatric condition, the impact of psychopharmacological treatment and somatic problems that interfere with cognition). This could complicate generalisation to other impaired populations (e.g. patients with brain injury or with milder psychiatric problems). Nevertheless, this sample can be seen as a realistic representation of patient groups that are met in clinical practice, and all included groups are equally concerned with these interfering factors. Second, methodological choices such as the cut‐off scores for the three ability groups were made based on the classification system of Resing and Blok (2002) and based on statistical favorability. One could argue that choices of cut‐off scores are in fact arbitrary and the usage of cut‐off scores (no matter which) will influence the results (Fogarty & Stankov 1995; Hartmann & Teasdale 2004; Hartmann & Teasdale 2005). These cut‐off scores resulted in a high difference in number of participants among the three groups (respectively 32, 78 and 140 subjects in the mild intellectual disability, borderline intellectual disability and normal/high intelligence groups). Furthermore, the choice to derive FSIQ scores from three intelligence test batteries as a measure of *g* instead of studying the association between Gf (e.g. perceptual reasoning index scores or WM index scores) and EF was both pragmatic and theory driven. Aligning three intelligence tests on the level of FSIQ scores results in a higher number of inclusions and in the highest reliable estimate of *g*. Although the construct of Gf strongly relates to *g* in both the Wechsler Scales – third and fourth editions, as well as in the KAIT, the amount of variance explained by Gf in the different index scores of the WAIS‐IV is lower than desired. Moreover, the subtests with a Gf‐measurement pretension have demonstrated higher factor loadings on visual processing than on Gf (van Aken *et al*. 2015). Third, as to statistical analyses, the power in this study may be regarded as insufficient for the within‐groups correlations and the ANCOVAs, reason why current results must be seen as preliminary, warranting further research with larger samples. In order to include more participants and facilitate analyses, the FSIQ scores of three intelligence tests were merged into one variable. Although clinicians should be careful in comparing IQ scores in individuals, the FSIQ scores of the used intelligence tests have shown to correlate highly with each other in research samples and can therefore be considered equally accurate and comparable measures of intelligence (Wechsler 2012c).

In sum, the correlational pattern of updating and intelligence, as well as the differential relation of shifting and intelligence across ability groups, suggests that EF tasks may not measure distinct EFs in lower intellectual ability but rely on cognitive primitives such as processing speed. Current neuropsychological tests may lack sufficient psychometric properties to adequately measure specific cognitive functions in low intellectual ability, and similar tasks used in all intellectual ranges call upon different cognitive abilities between different ability ranges. This hampers direct comparison of performance between low and high ability ranges. Given the high prevalence of premorbid intelligence deficits or ID in the (neuro)psychiatric population (Stratta *et al*. 2008; Verhoeven & Egger 2014; Pedersen *et al*. 2018), development of specific tasks is needed to disentangle *g* and specific domain‐dependent cognitive processes in this group.

## Conflict of interest

The authors declare no conflict of interest.
